# Improving the Predictive Value of Prion Inactivation Validation Methods to Minimize the Risks of Iatrogenic Transmission With Medical Instruments

**DOI:** 10.3389/fbioe.2020.591024

**Published:** 2020-12-01

**Authors:** Mohammed Moudjou, Johan Castille, Bruno Passet, Laetitia Herzog, Fabienne Reine, Jean-Luc Vilotte, Human Rezaei, Vincent Béringue, Angélique Igel-Egalon

**Affiliations:** ^1^Université Paris Saclay, INRAE, UVSQ, VIM, Jouy-en-Josas, France; ^2^Université Paris Saclay, INRAE, AgroParisTech, GABI, Jouy-en-Josas, France; ^3^FB.INT’L, Montigny-le-Bretonneux, France

**Keywords:** prion, sporadic CJD, amplification, PMCA, surface, decontamination

## Abstract

Prions are pathogenic infectious agents responsible for fatal, incurable neurodegenerative diseases in animals and humans. Prions are composed exclusively of an aggregated and misfolded form (PrP**^Sc^**) of the cellular prion protein (PrP^C^). During the propagation of the disease, PrP^Sc^ recruits and misfolds PrP^C^ into further PrP^Sc^. In human, iatrogenic prion transmission has occurred with incompletely sterilized medical material because of the unusual resistance of prions to inactivation. Most commercial prion disinfectants validated against the historical, well-characterized laboratory strain of 263K hamster prions were recently shown to be ineffective against variant Creutzfeldt-Jakob disease human prions. These observations and previous reports support the view that any inactivation method must be validated against the prions for which they are intended to be used. Strain-specific variations in PrP^Sc^ physico-chemical properties and conformation are likely to explain the strain-specific efficacy of inactivation methods. Animal bioassays have long been used as gold standards to validate prion inactivation methods, by measuring reduction of prion infectivity. Cell-free assays such as the real-time quaking-induced conversion (RT-QuIC) assay and the protein misfolding cyclic amplification (PMCA) assay have emerged as attractive alternatives. They exploit the seeding capacities of PrP^Sc^ to exponentially amplify minute amounts of prions in biospecimens. European and certain national medicine agencies recently implemented their guidelines for prion inactivation of non-disposable medical material; they encourage or request the use of human prions and cell-free assays to improve the predictive value of the validation methods. In this review, we discuss the methodological and technical issues regarding the choice of (i) the cell-free assay, (ii) the human prion strain type, (iii) the prion-containing biological material. We also introduce a new optimized substrate for high-throughput PMCA amplification of human prions bound on steel wires, as translational model for prion-contaminated instruments.

## Introduction

Transmissible spongiform encephalopathies (TSE) or prion diseases are fatal, uncurable neurodegenerative diseases affecting animals and humans (Collinge, 2001). TSE include scrapie in sheep and goats, bovine spongiform encephalopathy (BSE), chronic wasting disease in cervids and Creutzfeldt-Jakob disease (CJD) in humans. Intra- and inter-species TSE transmission has recurrently occurred in animals and humans via medical and dietary settings. BSE has occurred as an epidemic in cattle and has propagated in human under the form of variant CJD (vCJD). Other dietary exposure in human includes kuru epidemic among Fore people of Papua New Guinea due to funerary cannibalism. Iatrogenic CJD forms are related to the use of contaminated cadaver-extracted human growth hormone and dura mater or to insufficiently sterilized contaminated brain surgery material. The most common forms of CJD are inherited or sporadic.

Transmissible spongiform encephalopathies are caused by prions. Prions are unconventional pathogens exclusively composed of an aggregated and misfolded form (PrP**^Sc^**) of the cellular prion protein (PrP^C^). During the disease pathogenesis, PrP^Sc^ recruits PrP^C^ and induces its misfolding into further PrP^Sc^. This replicative self-templating process is at the origin of prion infectious nature (Prusiner, 1982). Biochemically, PrP^Sc^ and PrP^C^ properties strongly differ. PrP^Sc^ is β-sheet rich, contains a protease-resistant core and is prone to aggregation, while PrP^C^ is α-helix rich, protease-sensitive and monomeric (Colby and Prusiner, 2011).

Susceptible mammals, including laboratory species, stably propagate structurally distinct PrP^Sc^ assemblies known as prion strains. Prion strains differ biochemically at the level of PrP^Sc^ tertiary and quaternary structures. Phenotypically, prion strains encode unique stereotypical biological traits including the time course to disease, neuropathological features and tropism for specific brain regions or lymphoid organs (Bruce, 2003; Collinge and Clarke, 2007; Beringue et al., 2008b; Weissmann et al., 2011). Co-propagation of strains has been observed, notably in CJD (Cassard et al., 2020) and sheep scrapie (Le Dur et al., 2017; Huor et al., 2019). PrP^Sc^ structural polymorphism is mostly considered as between strain polymorphism. However, experimental evidence supports the view for further structural diversity and co-propagation of PrP^Sc^ assemblies within specific prion populations and strains (Igel-Egalon et al., 2019a).

While human TSE remain relatively rare, they constitute a critical public health concern. First, the disease incubation period is long, asymptomatic, without impact on most biological constants. Early diagnosis of the disease is lacking. Medical, non-disposable instruments may thus be used on individuals incubating silently the disease and potentially be reused. Second, while prions are neurotropic agents, they can replicate extraneurally at significant levels, markedly increasing the range of medical acts that can transmit the disease iatrogenically. Third, prions are highly resistant to common inactivation methods as compared to viruses or bacteria. Since 1999, the World Health Organization guidelines and their national counterparts recommend procedures, which include immersion of non-disposable surgical instruments in 1M sodium hydroxide (NaOH) or 20,000 ppm sodium hypochlorite (2% NaOCl) for 1h, followed by porous autoclaving at 134°C for 18 min (WHO, 1999; DGS, 2011). To circumvent the limits imposed by these methods (e.g., instrument corrosion), the French Medicine Agency (ANSM), for example, recommend since 2011 a list of commercial methods validated for their efficacy to inactivate prions according to a standardized protocol (Ansm, 2011). The validation studies were exclusively based on the inactivation of laboratory prion strains, including as primary model with short incubation time and high infectivity titer hamster-adapted scrapie 263K (or Sc237) prions (Kimberlin and Walker, 1977), as model relevant to BSE/vCJD, mouse-derived BSE prions (Bruce et al., 1994; Lasmezas et al., 1996) or Fukuoka-1 prions derived from the mouse adaptation of human, inherited Gerstmann-Sträussler-Scheinker (GSS) syndrome (Tateishi et al., 1979) as model relevant to human TSE. The inactivation of these prion strains was primarily tested by measuring residual infectivity in hamster or mouse bioassays. However, prion inactivation efficacy is strain-dependent. For example, sporadic CJD prions were shown to be 100,000-fold more resistant than hamster Sc237 prions to acidic SDS treatment (Peretz et al., 2006). The host species in which the strain is passaged can also impact the final efficacy. For example, cattle BSE prions were >1,000-fold more resistant to acidic SDS treatment than BSE-derived mouse 301V prions (Giles et al., 2008). Extended heating or steam sterilization by autoclaving similarly inactivated prions in a strain-dependent manner, BSE and BSE-derived sources being amongst the most resistant strains (Fernie et al., 2012; Marin-Moreno et al., 2019). Worryingly, most ANSM-validated disinfectants which totally inactivated 263K, mouse-BSE or Fukuoka-1 prions were subsequently shown to partially inactivate human vCJD prions (Belondrade et al., 2016; Bélondrade et al., 2020). Collectively, these observations strongly support the view that any inactivation method must be validated against the prions for which they are intended to be used.

The studies by Belondrade et al. (2016); Bélondrade et al. (2020) suggested that cell-free prion amplification assays may replace animal bioassays to quantify prion inactivation efficacy. The European and French Medicine agencies thus implemented their protocols to validate prion inactivation methods. They encourage or request the use of both human prions (or human-relevant prions) and highly sensitive cell-free prion amplification assays (Ansm, 2018; EMA, 2018). These assays measure prion concentration by limiting dilution titration, based on PrP^Sc^ seeding activity. Their sensitivity is equivalent or greater than animal bioassays which are measuring prion infectivity. Yet, they are not routinely used in inactivation methods. In this review, we discuss the methodological and technical issues raised by such implementations, with respect to the choice of the cell-free assay, the human prion strain type and the nature of the prion-containing biological material.

## Replacing Animal Bioassays by Cell-Free Assays in Prion Inactivation Methods

**FIGURE 1:**
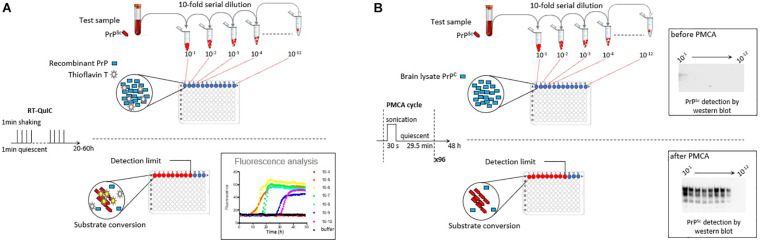
Scheme summarizing the principles of the RT-QuIC and PMCA assays. **(A)** RT-QuIC is based on the polymerization of recombinant PrP protein by prions in the test sample. During iterative cycles of shaking and quiescent incubation, polymerization is monitored in real-time by the increase in Thioflavin T fluorescence. **(B)** PMCA is based on the bona fide conversion of brain PrP^C^ by prions in the test sample. Conversion is favored by iterative cycles of quiescent incubation and sonication. At the end of the reaction, the presence of abnormal PrP^Sc^ is analyzed by proteinase K digestion and western blot. In both methods, prion seeding activity in the test sample can be quantified by limiting dilution titration.

Measuring prion concentration in a test sample has for long relied on measuring prion infectivity by bioassay in laboratory animals. Prion infectious titer can be obtained by endpoint dilution titration of the sample in bio-indicator animals or by using incubation time values once prion dose response curves have been established (Prusiner et al., 1980, 1982). Surrogately, cell-free assays estimate prion concentration by measuring prion self-converting activity. Among them, two ultrasensitive amplification assays deserve attention to their effectiveness to detect minute amounts of prions: the real-time quacking-induced conversion (RT-QuIC) assay (Atarashi et al., 2008; Wilham et al., 2010) and the protein misfolding cyclic amplification (PMCA) assay (Saborio et al., 2001; Figure 1). In essence a PrP^Sc^-containing sample is mixed with a substrate containing normally folded, monomeric recombinant PrP (RT-QuIC) or brain PrP**^C^** (PMCA). The conversion is favored and accelerated by cycles of shaking (RT-QuIC) or sonication (PMCA) and quiescent incubation. In the RT-QuIC assay, the conversion of recombinant PrP into amyloid aggregates is followed in real-time by incorporation of thioflavin T (ThT), an amyloid-sensitive fluorescent dye. In the PMCA assay, the conversion of PrP**^C^** into PrP**^Sc^** is assessed at the end of the reaction by biochemical purification and immunodetection of PrP^Sc^. Both tests reach similar or greater sensitivities than those of the animal bioassays. Both have a wide range of fundamental and applied applications, including TSE diagnostic. Both have benefits and drawbacks that make them complementary techniques in the TSE field, as summarized in Table 1.

**TABLE 1:** Benefits and drawbacks of the RT-QuIC and PMCA assays.

	RT-QuIC	PMCA
Substrate	Recombinant PrP	Cellular form of PrP
Protocol	Shaking/incubation	Sonication/incubation
Measure of prion concentration	PrP^Sc^ seeding activity	PrP^Sc^ seeding activity
Conversion products	Thioflavin T positive amyloid PrP	PrP^Sc^ assemblies
Pros	-Real time and direct readout -Low biohazard -Detection in bodily fluids, notably CSF -CSF-based diagnostic for CJD -Commercial substrate available -Universal substrate -Good inter-laboratory reproducibility of the second-generation RT-QuIC -Use in other neurodegenerative diseases	-Generation of bona fide prion infectivity -Prion strain typing -Detection of sub-infectious prion doses -Detection in bodily fluids, notably blood -Correlation between infectivity and seeding activity in inactivation tests
Cons	-Amplified product poorly infectious -Discrepancies between infectivity and seeding activity in inactivation tests	-Indirect readout (immunoblot) -No universal substrate -Strains refractory to amplification -Biohazardous method -No commercial substrate -Limited inter-laboratory reproducibility -Use of cofactors required for certain strains
Application	Diagnostic	Research use

### The RT-QuIC Assay

In the RT-QuIC assay, the ThT-positive amyloid assemblies formed by seeded recombinant PrP are poorly infectious (Groveman et al., 2017; Raymond et al., 2020). Manipulation of prion infectivity is thus limited to the seed, limiting biohazards in routine use of the assay. A limiting aspect of RT-QuIC is the possible self-polymerization of recombinant PrP in the absence of any seed. From a diagnostic viewpoint, RT-QuIC is particularly efficient at amplifying prion sub-infectious doses in sample biopsies from the skin (Orru et al., 2017), olfactory mucosa (Orru et al., 2014), urine, saliva (Henderson et al., 2015), blood (Elder et al., 2013), and CSF (McGuire et al., 2012). The second generation of RT-QuIC specially designed for prion detection in the CSF achieved >95% sensitivity and 100% specificity (Orru et al., 2015a), with a good interlaboratory reproducibility, thus opening new avenue for ante-mortem diagnosis of CJD (Rhoads et al., 2020). CSF-based RT-QuIC is now considered as the most powerful versatile technique for diagnosing CJD. It is recommended by the American Center for Disease Control and Prevention (CDC) since 2018. Following this pioneering work, similar CSF-based assays were successfully developed for other prion-like neurodegenerative diseases linked to protein misfolding. These RT-QuIC assays identify alpha-synuclein (Rossi et al., 2020) and Tau (Kraus et al., 2019; Saijo et al., 2020) seeding activity in CSF of patients with Lewy bodies or Parkinson’s disease and Alzheimer’s disease or frontotemporal lobar degeneration, respectively. Proof of concept has been obtained with TDP-43 in amyotrophic lateral sclerosis and frontotemporal lobar degeneration (Scialò et al., 2020).

Given the analytical sensitivity of the RT-QuIC assay and the clear correlation between seeding activity and infectivity in measuring prion concentration (Wilham et al., 2010), its potential to assess prion inactivation methods was assayed. There were discrepancies between the animal bioassay and the RT-QuIC assay. One disinfectant inactivating all measurable prion infectivity by bioassay was not able to eliminate all seeding activity by RT-QuIC (Hughson et al., 2016). In another study, the seeding activity of human CJD prions bound to stainless steel wires was measurable by RT-QuIC and completely removed after a 2 h-treatment with 1M NaOH (Mori et al., 2016), as expected. The reason for such discrepancies is unclear. Prion inactivation treatment may destroy prion infectivity without affecting all PrP seeding activity, given the high sensitivity of the assay. Alternatively, as the RT-QuIC generates amyloid fibers that are off-pathway to prion infectivity, treatment neutralizing these forms may not necessarily affect prion infectivity *per se*.

### The PMCA Assay

As key feature, PMCA mirrors in an accelerated manner the prion replication process (Igel-Egalon et al., 2019b). The reaction products are highly infectious – with infectivity titers equivalent to those found in the brain at the terminal stage of the disease – and contain prions which generally retain the parental seed strain phenotype (Weber et al., 2007; Shikiya and Bartz, 2011; Moudjou et al., 2014). PMCA has been instrumental in identifying co-factors (Deleault et al., 2012a,b; Fernández-Borges et al., 2018), PrP domains (Burke et al., 2020a) or post-translational modifications (Moudjou et al., 2016; Burke et al., 2020b) involved in the prion replication process. It has also been instrumental in studying the diversification of PrP^Sc^ assemblies and their dynamic interactions (Igel-Egalon et al., 2019b).

The analytical sensitivities of the PMCA and RT-QuIC assays are generally equivalent. RT-QuIC is the method of choice to quantify the seeding activity of prions responsible for sporadic CJD (notably the most prevalent MM1 subtype), whereas PMCA is more performant at detecting vCJD prions (Moudjou et al., 2014; Camacho et al., 2019). PMCA has been incredibly good at detecting vCJD/BSE prions in blood from asymptomatic carriers, in small and large animal models and in human (Lacroux et al., 2014; Bougard et al., 2016; Concha-Marambio et al., 2016). Yet, the necessity to work with PrP^C^-containing cell or brain substrate and the absence of real-time detection of the conversion process are major impediments to a diagnostic use. Several laboratories are working at substituting PrP^C^-containing brain substrate with recombinant PrP (Fernández-Borges et al., 2018; Eraña et al., 2019).

With respect to prion inactivation methods, a strong correlation between animal bioassay and PMCA was found in two compelling studies testing several disinfectants or physical processes on 263K prions or vCJD prions bound on stainless steel wires (Pritzkow et al., 2011; Belondrade et al., 2016; Bélondrade et al., 2020). PMCA thus emerged as a potent tool to evaluate the degree of effectiveness of prion inactivation methods, at least with certain human and laboratory strains.

## Finding the Right Prion Strain in the Right Shape to Standardize Prion Inactivation Methods

### Human Prion Strains

The choice of the “right” human prion strain to implement the validation of prion inactivation methods is a conundrum:

1. None of the human prion strains have been studied as deeply as hamster 263K with respect to pathogenesis, infectivity titers and response to inactivating treatments by bioassay;
2. The use and relevance of human prions adapted to wild-type laboratory animals must be studied on a case-by-case basis. For example, mouse-adapted Fukuoka-1 prions were not able to propagate on back-passage to transgenic mice expressing human PrP, suggesting loss of parental CJD transmission characteristics (Giles et al., 2017). Oppositely, sporadic CJD prions propagated in bank voles with little or no species barrier, consistent with potential maintain of CJD transmission characteristics (Nonno et al., 2006). Early transmission studies indicated that non-human primates were susceptible to sporadic and vCJD prions (Gibbs et al., 1968; Lasmézas et al., 1996), providing a potential source of human-like prion infectivity. However, macaque and human PrP sequences differ by nine amino acids, which were shown by transgenic modeling to create a substantial species barrier on transmission of sporadic CJD (Espinosa et al., 2019). Whether macaque-passaged prions retain *in fine* CJD parental properties remain to be determined. Seminal studies by transgenic modeling indicated that amino acid sequence identity between PrP^Sc^ and PrP^C^ usually abrogate the barrier to transmission between species (for review Moreno and Telling, 2017). Human prions did not escape the rule. Sporadic and vCJD prions propagate without species barrier in mice transgenic for human PrP, in the absence of mismatch at polymorphic codon 129 (Bishop et al., 2010; Beringue et al., 2012; Chapuis et al., 2016; Jaumain et al., 2016; Cassard et al., 2020). These models, – in the absence of cell models propagating human prions –, allow a relatively rapid, inexpensive, and large production of potentially biologically cloned and well-characterized *humanized* prions with respect to pathogenesis, strain type and infectivity titers. Oppositely, sporadic and vCJD reference reagents from the World Health Organization contain mixture of strains (Minor et al., 2004; Beringue et al., 2008a; Yull et al., 2009), which may complicate their use in decontamination protocols. It remains to be determined whether *humanized* prions exhibit similar resistance/sensitivity to inactivation as those that accumulate directly in the human brain. At least, they seem to share similar levels of infectivity (Beringue et al., 2008a; Halliez et al., 2014);
3. The pros and cons of assaying (human or *humanized*) sporadic or vCJD prions in inactivation trials must be weighed. While the number of clinical cases of vCJD has remained limited (∼235 cases worldwide), this disease is a key issue due to the large number of potential asymptomatic carriers (Gill et al., 2020), the large distribution of infectivity in the body (Douet et al., 2017) and the associated risks of secondary transmission, notably by blood transfusion or surgery (Peden et al., 2004). The disease is due to a unique prion strain type (classical BSE prions) (Diack et al., 2012). When testing heat sterilization methods, BSE/vCJD prions thermostability may increase the predictive value of the assay (Fernie et al., 2012; Marin-Moreno et al., 2019). Sporadic forms of CJD are more prevalent with approximately 1.5 cases per millions per year. The disease is heterogeneous, making the choice of the “right” strain a dilemma; nine sub-types of sporadic CJD are described according to the clinical and neuropathological characteristics of the disease in infected individuals, the polymorphism of the *PRNP* gene at codon 129 (MM, MV, or VV) and the electrophoretic profile of brain PrP^Sc^ (type 1 or type 2) (for review Zerr and Parchi, 2018). At least six distinct strain types are described according to their transmission properties in transgenic mice expressing human PrP. Starting from the most prevalent cases in the population, these strains are classified as MM1/MV1, VV2, MV2, VV1, cortical-MM2, thalamic-MM2 strains (Bishop et al., 2010; Moda et al., 2012; Chapuis et al., 2016; Jaumain et al., 2016; Cassard et al., 2020). Co-propagation of MM1 and VV2 strains has been observed in a significant proportion of sporadic CJD patients (Cassard et al., 2020).

Although direct assay of human prions in inactivation methods may sound highly relevant with respect to iatrogenic risk of prion transmission, it necessitates a thorough examination to ensure that the results obtained are of added value and that extrapolation to the human situation can be made.

### Stainless Steel Wires as Model for Surgery Instruments

Prions and bacterial biofilms are a challenge to proper sterilization of non-disposable medical devices because of their high resistance to inactivation and binding affinity for steel surfaces. To date, six cases of iatrogenic CJD have been reported worldwide by contaminated surgical instruments or depth EEG electrodes (for review Bonda et al., 2016). Prion-contaminated stainless steel wires have become the gold standard to screen and validate prion inactivation methods for medical instruments. This translational model, which is used in most guidelines, came from the seminal work of Charles Weissmann and collaborators (Zobeley et al., 1999; Flechsig et al., 2001). In essence, wires are artificially contaminated in prion-containing brain macerates. To measure prion concentration pre- and post-disinfection, the wires are permanently implanted in the brain of bio-indicator animals and reduction in the disease attack rate is measured. The reduction factor is established by comparing the results obtained with endpoint titration of prions bound on steel wires. A prion-cell endpoint assay (Klohn et al., 2003) was adapted that offered greater sensitivity, practicability and rapidity than the animal bioassay (Edgeworth et al., 2009). However, its use is so far limited to mouse prions. A sensitive assay based on direct immunodetection of surface-bound prions was also reported and validated against human vCJD prions (Edgeworth et al., 2011b).

Key issues in the interpretation and overall validity of the results are the rate of prion adsorption, desorption and/or bio-activity (i.e., ability to initiate infection in the brain). With mouse or hamster prions, a contact of a few minutes between the infected brain homogenate and the wire was sufficient to transmit the disease with 100% attack rate (Flechsig et al., 2001; Giles et al., 2017). A transient insertion of contaminated wires in the brain for 5–30 min was sufficient to transmit the disease to laboratory animals with 100% attack rate, yet with delayed disease tempo (Flechsig et al., 2001; Yan et al., 2004). This suggested relatively rapid rate of adsorption and release/bio-activity. The information on prion fate once surface-adsorbed is relatively limited. Binding to soil fractions (montmorillonite) was reported to potentiate the disease transmission capacity of hamster 263K prions by oral route (Johnson et al., 2007). The opposite was found on binding to silty clay upon intracerebral inoculation (Saunders et al., 2011). In “standard” conditions, wires are contaminated for 1 h and permanently inserted in bio-indicator animals. To further circumvent these uncertainties and address the possibility that the decontamination procedures unbound prions from the wires without inactivating them, an additional study on the inactivation potential of the method on desorbed material may be requested (Ansm, 2011, 2018).

### Heterogeneity of Prion Assemblies

The brain tissue does not represent the more likely source of iatrogenic prion contamination, except during neurosurgical procedures. The use of sensitive animal bioassays with human PrP transgenic mice and/or cell-free assays allowed demonstrating that many tissues outside the brain contain substantial amounts of prions, thus markedly increasing the range of medical acts with non-disposable equipment that may transmit human TSE iatrogenically. This includes dentistry, organ transplant, blood transfusion, and surgery. In individuals infected with vCJD, at the symptomatic or pre-symptomatic stage, prions have been detected in a wider and more unexpected variety of peripheral tissues (Douet et al., 2017) than previously reported (Wadsworth et al., 2001). Those include bone marrow, kidney, salivary glands, skeletal muscle, pancreas, liver or heart in addition to tissue of the lymphoid system. In sporadic CJD, while prion distribution is more intense in the central and peripheral nervous systems, substantial amounts of prions have been found in the bone marrow (Huor et al., 2017), skin (Orru et al., 2017), kidney, lung, liver, adrenal glands (Takatsuki et al., 2016), and muscle (Peden et al., 2006; Rubenstein and Chang, 2013). Prions were also detected in biological fluids, notably blood, and urine of patients with sporadic and variant of CJD, often well before the onset of early clinical signs (Edgeworth et al., 2011a; Lacroux et al., 2014; Moda et al., 2014; Sawyer et al., 2015; Luk et al., 2016). Confirmed cases of iatrogenic transmission of vCJD by blood transfusion, and a probable case in a patient treated with coagulation factors VIII manufactured from plasma indicate that blood constitutes an effective source of iatrogenic contamination (Peden et al., 2004, 2010; Wroe et al., 2006). It was reported that the prion protein PrP^C^ is a major contaminant of the purified urinary-derived gonadotropins used in infertility treatment (Van Dorsselaer et al., 2011). These elements raise specifically the question of prion biosafety of blood, blood-derived products, and urine-derived drug products. Analytical technics for securing drug manufacturing process from blood and urine must be reconsidered and implemented as those securing medical instrumentation, as recently recommended by the European Medicines Agency (EMA, 2018).

Prion presence in several tissues and bodily fluids raises the question of the most physiologically relevant PrP^Sc^ aggregates and of the best experimental approach for their inactivation. Most studies use brain macerates or microsomal brain fractions to contaminate steel wires or as spike for studying prion removal. Their high infectious titer compared to other extraneural tissues or fluids obviously increases the analytical sensitivity of the approach. However, PrP^Sc^ assemblies in bodily fluids or extraneural tissue may be of smaller size than in the brain. Compelling evidence indicate that different subassemblies with specific structural properties are co-accumulating in the brain at the disease terminal stage. These sub-assemblies harbor different size, different infectivity titer or seeding activity (for review Igel-Egalon et al., 2019a). Their capacity to bind steel wires or their retention properties may vary.

Further, the preparation of the spike or of the dilutions in the *ad hoc* experimental conditions may modify PrP^Sc^ assembly composition. In our previous work, we demonstrated that PrP^Sc^ assemblies have two levels of organization (Igel-Egalon et al., 2017). The first one is formed by the packing of oligomeric building blocks (called suPrP) into larger assemblies. In this organization, the cohesion forces are weak, as shown that rapid depolymerization following urea chaotropic treatment. The second level of organization is formed by suPrP itself. suPrP is a very stable oligomer between a dimer or a tetramer that resists >6–8 M urea. This study led us to conclude that PrP^Sc^ assemblies and their building blocks are in a highly dynamic equilibrium (Igel-Egalon et al., 2017, 2019a). A simple dilution of purified PrP^Sc^ assemblies was able to drive the equilibrium towards their depolymerization into suPrP. A dilution process may thus drive which PrP^Sc^ morphotype is submitted to the inactivation/retention process; in return PrP^Sc^ morphotype are likely to react to such process in a structural-dependent manner. To conciliate the advantage of using brain extract with the relevance of the model, one alternative could be the use of small PrP^Sc^ particles, obtained by fractionation experiments. We previously demonstrated that these assemblies have high specific infectivity values despite their small size (Tixador et al., 2010; Laferriere et al., 2013) and are relatively stable over time out of the conversion process (Igel-Egalon et al., 2019b).

As for the nature of the prion strain, these data collectively support the view that the retention/inactivation methods must be validated against the biological material for which they are intended to be used.

## Human Prion PMCA

### PMCA Improvements and Putative Mechanisms of Prion Amplification

Efficient amplification of human prions by PMCA is strain dependent. It was initially believed that optimal amplification of human prion strains required absence of mismatch at codon 129 between the seed and the substrate. Thus, Jones et al. (2009) classified CJD subtypes in two distinct groups according to their “preference” for the *PRNP* genotype substrate. The first group, composed of vCJD, MM1, MM2, and MV1 sporadic prions, is preferentially amplified by the *PRNP*-129MM substrate. The second one, composed of VV1, VV2, and MV2 sporadic CJD prions, is more efficiently amplified by *PRNP*-129VV substrate. Despite this sequence compatibility, prions were not amplified to a degree of sensitivity sufficient for validating decontamination methods.

Other parameters can be adjusted to improve PMCA efficacy: (1) Obviously, the number of PMCA rounds can be increased. While it was initially suspected to increase the probability of de novo PrP^Sc^ formation in the absence of preexisting prions, carefully designed PMCA operating conditions suggested that spontaneous prion formation in PMCA reactions was rather a result of inadvertent cross-contamination (Cosseddu et al., 2011). Three to six rounds of PMCA are routinely used to amplify low amounts of human prions (Lacroux et al., 2014; Bougard et al., 2016; Concha-Marambio et al., 2016; Cassard et al., 2020); (2) The PrP^C^ substrate can be optimized. The higher the PrP^C^ concentration, the higher the sensitivity achieved (Mays et al., 2009; Moudjou et al., 2014, 2016), which lends support for the use of brain from transgenic mice overexpressing PrP^C^. Further, markedly improved sensitivities, including with human prions, were obtained when PMCA was performed with partially desialylated (Katorcha et al., 2015) or unglycosylated PrP^C^ (Nishina et al., 2006; Camacho et al., 2019) as substrate. The authors attribute this phenomenon to sialic acid electrostatic repulsion forces and the glycan steric hindrance that may interfere with the conversion of PrP^C^ by PrP^Sc^. Whether this would work with all strains given their variable glycoform requirements (Khalili-Shirazi et al., 2005; Nishina et al., 2006) remains to be determined; (3) Conversion enhancers can be added to the PMCA reaction, including polyanions (Fernández-Borges et al., 2018), anionic lipids (Deleault et al., 2007), or dextran sulfate (Moudjou et al., 2016). Their mode of action on the conversion process remains poorly understood. Their impact on ionic strength, osmolarity, water molecule organization, PrP^C^ or assemblies stability may play a role in the replication/templating process; (4) Physical change in the PMCA environment such as the reaction volume or the addition of microbeads in the substrate affected significantly the amplification efficacy with respect to the dilution achieved (Gonzalez-Montalban et al., 2011; Johnson et al., 2012; Moudjou et al., 2014).

The commonly shared view that microbeads addition increases the fragmentation of the newly converted PrP^Sc^ assemblies during the sonication steps and therefore multiplies the number of templating interface for conversion was recently challenged. By using sedimentation velocity to explore the quaternary structure of prion assemblies, we found no evidence for fragmentation in PMCA conditions with beads (Igel-Egalon et al., 2019b). Alternative hypotheses such as beads serving as thermoacoustic convertor (Povey et al., 2011) and/or as multidirectional secondary source may be considered.

**FIGURE 2:**
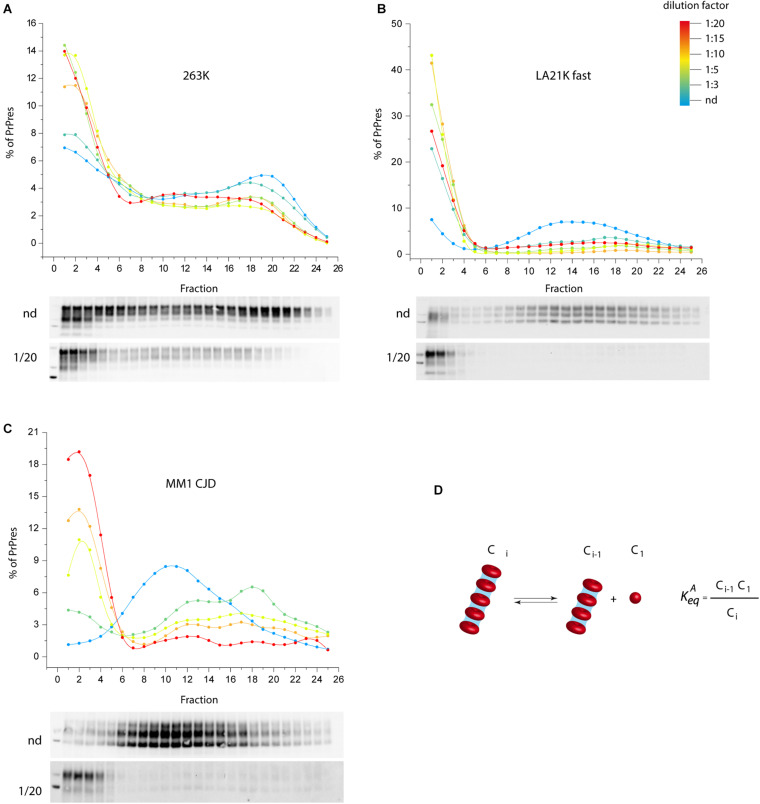
The disassembling effect of PMCA buffer on PrP^Sc^ assemblies. **(A–C)** Twenty percent infected brain homogenate from hamster 263K prions (Kimberlin and Walker, 1977), sheep-derived LA21K *fast* (Le Dur et al., 2017), and *humanized* MM1 sporadic CJD prions (Beringue et al., 2008a) were directly diluted [not diluted (nd), 1:3, 1:5, 1:15, 1:20 from blue to red line] in a PrP^0/0^ lysate made in PMCA buffer (thus in the absence of PrP^C^ substrate). After a 4 h-incubation at 4°C in the absence of sonication, PrP^Sc^ assemblies quaternary structure was investigated by sedimentation velocity fractionation (Tixador et al., 2010). The collected fractions (numbered from top to bottom of the gradient) were analyzed for PrP^Sc^ content by immunoblot. The graphs show the relative amount of PrP^Sc^ per fraction. Representative immunoblots are shown. For the three prion strains, dilution induced a disassembling process generating small PrP^Sc^ objects. **(D)** PrP^Sc^ assemblies (C_*i*_) dilution in PMCA buffer tends to displace a detailed-balance toward the formation of smaller objects here termed C_1_, which are assimilated to suPrP (Igel-Egalon et al., 2017).

We propose an alternative model to prion fragmentation to explain prion amplification during PMCA, based on the existence of a detailed-balance between PrP^Sc^ assemblies and suPrP (Chyba et al., 2020). A perturbation in prion environment would shift the balance towards PrP^Sc^ assemblies depolymerization into suPrP, thus multiplying the number of templating interface. We experimentally investigated this by studying the impact on PrP^Sc^ assemblies size of diluting infected brain homogenate in PMCA buffer, out of a replicative context and without sonication. As shown in Figures 2A–C, a >1:3 dilution was sufficient to depolymerize PrP^Sc^ assemblies into smaller objects, indicating an equilibrium displacement (Figure 2D). This was observed for three different prion strains, suggesting a possible generic effect. This observation supports the view that the physico-chemical properties of the PMCA buffer are enough to disrupt PrP^Sc^ assemblies, suggesting therefore that PrP^Sc^ fragmentation does not occur during bead-PMCA reactions.

Despite these major improvements, certain prion types remain unamplifiable by PMCA or do not achieve degrees of amplification requested by the medicine agencies (6 Log_10_ of magnitude (Ansm, 2018)). The MM1 subtype remains the most difficult sporadic CJD subtype to amplify. At variance with RT-QuIC (Orru et al., 2015b), there is no universal PMCA substrate for human CJD prions.

### A PMCA Substrate to Amplify With High Sensitivity Human Prion Strains Adsorbed on Steel Surface or in Suspension

**FIGURE 3:**
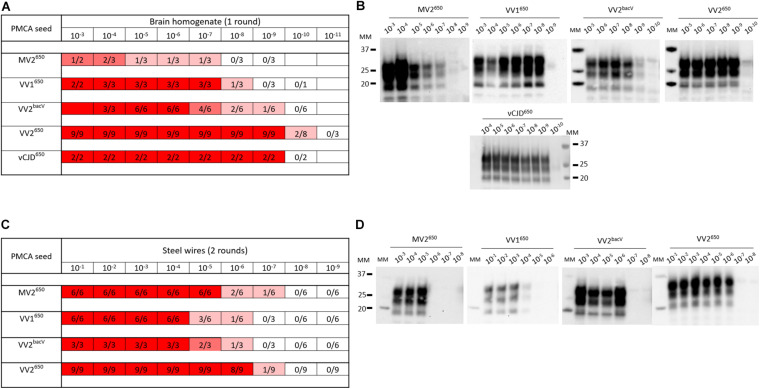
PMCA detection of sporadic and variant CJD prions in suspension or bound to steel wires using BacV-derived human PrP substrate. Serial dilutions of *humanized* sporadic CJD subtypes and variant CJD (vCJD) prions passaged into human PrP mice [tg650 line (Met129 allele) or BacV line (Val129 allele)] were used in suspension **(A,B)** or bound to steel wires **(C,D)**. They were mixed with uninfected BacV brain homogenate and submitted to the mb-PMCA for one round **(A,B)** or two rounds **(C,D)** of 96 cycles of sonication/incubation for 48 h. The PMCA products were analyzed for PrP^*Sc*^ content by immunoblot. **(A,C)** Summary of the results obtained. The red boxes indicate the presence of PrP^*Sc*^ in PMCA products, the ratio in each box corresponds to the number of PrP^*Sc*^-positive amplification products over the number of PMCA replicates performed. **(B,D)** Representative immunoblots (Sha31 anti-PrP antibody). MM, molecular weight markers.

With all the potential factors improving PMCA sensitivity in mind, we optimized a substrate to amplify human sporadic CJD prions. First, we designed a new transgenic mouse line homozygous at the locus transgene that overexpressed approximately five- to sixfold the valine allele at codon 129 of human PrP^C^ (BacV line) on a pure FVB/N PrP-knockout background (Passet et al., 2020). The transgene designed for targeting expression of human PrP in these mice is based on a large human BAC insert (the details of the transgenic mice will be published elsewhere). The brain of these mice was used as PrP^C^ substrate in PMCA reactions (see Supplementary Material for the methods section). To improve the sensitivity of the reaction, we added polymers of dextran, one teflon microbeads and worked with a reduced reaction volume, as previously described in our so-called mb-PMCA protocol (Moudjou et al., 2014, 2016). As seed, we used brain homogenates from *humanized* mice in which we isolated and phenotypically characterized different sporadic CJD subtypes (Jaumain et al., 2016). Those were serially diluted and submitted to one to three rounds of mb-PMCA reaction. *Humanized* MV2, VV1, VV2 sporadic CJD prions, and vCJD prions were used. Positive reactions were obtained for these *humanized* strain types up to the 10^–7^ (MV2 subtype) to 10^–10^ (VV2 subtype) dilution (Figures 3A,B). Noticeably, mismatch at codon 129 between seed and substrate was not detrimental as the highest levels of amplification were observed with sporadic CJD VV2 prions and vCJD prions serially passaged onto transgenic mice expressing human-PrP^*C*^ with Met at codon 129. The dynamic of amplification obtained with vCJD prions (limiting dilution at 10^–9^) or VV2 (10^–9^ or 10^–10^ depending on the allele on which the subtype was propagated) offered an analytical sensitivity compatible with the validation of prion inactivation methods. In the case of vCJD prions, it was more sensitive than animal bioassays by at least two orders of magnitude (Douet et al., 2014; Halliez et al., 2014).

In the context of the risk evaluation associated with the decontamination of medical instruments, we further evaluated the effectiveness of the BacV-derived mb-PMCA substrate to detect human prions bound onto steel surface. Briefly, fine stainless steel wires were contaminated with serial dilutions of prion-containing brain homogenate (Zobeley et al., 1999; Flechsig et al., 2001) and added directly to the mb-PMCA reaction mix as seed. As summarized in Figures 3C,D, two rounds of PMCA were enough to detect bound prions up to the 10^–6^–10^–7^ dilution. Again, these limiting dilution values were in the range required for appraisal of inactivation methods by using steel wire as translational model for medical instruments.

## Conclusion

This mini-review addresses the questions raised by the implementations, by the competent authorities, of prionicidal product authorizations. Any validation procedure should be tested against prions for which the inactivant is intended to be used, suggesting that human or *humanized* prions should be used in fine. However, several pending questions are emerging, including the choice of the prion subtype and of the prion-containing biological matrix. Another operational aspect to consider is the high biohazard level to manipulate these agents as compared to laboratory 263K prions.

Given the correlation between PMCA and animal bioassay in measuring prion concentration bound on steel wires or in suspension, there is a proof of concept that this cell-free assay could complement and even replace animal bioassays. This would provide an economical and ethically sound method. Yet, certain human prion subtypes remain poorly amplifiable, limiting the potential relevance of the assay. Given the high-throughput, rapid format of the assay, the use of several prion subtypes may circumvent this. Animal bioassays using human or *humanized* prion are still considered as gold standard methods. These models and the PMCA assay are mostly manipulated in academic laboratories and not in Contract Research Organizations (CROs) that routinely perform studies for biocidal products authorizations. This may delay the time to market of these products.

## Author Contributions

VB, HR, and AI-E designed the study, analyzed the data and drafted the manuscript. J-LV, BP, JC, and VB designed the new mice line. AI-E, MM, LH, and FR performed the experiments. All authors gave their final approval of the submitted version.

## Conflict of Interest

The authors declare that the research was conducted in the absence of any commercial or financial relationships that could be construed as a potential conflict of interest.
