# Supplementary material for: Improving the Predictive Value of Prion Inactivation Validation Methods to Minimize the Risks of Iatrogenic Transmission With Medical Instruments
Source: Front Bioeng Biotechnol. 2020 Dec 1;8:591024. doi: 10.3389/fbioe.2020.591024 (PMC7736614; doi:10.3389/fbioe.2020.591024)
Supplement: Supplementary file 1 [file Data_Sheet_1.DOCX]

Supplementary Material

# Ethics statement

All animal experiments were approved by the INRAE/AgroParisTech Ethics Committee (Comethea; permit number 15/056). Mouse euthanasia was performed by cervical column disruption. Brains were rapidly removed and kept at -80°C for further analysis.

# Transgenic mice lines and prion sources

sCJD-VV2, -VV1, -MV2, -MM1, -MV1 and vCJD *humanized* prion strains are experimentally cloned prions strains obtained by serial transmission of human cases to tg650 transgenic mice expressing the Met129 allele of human PrP (Béringue *et al.*, 2008; Jaumain *et al.*, 2016). The LA21K *fast* scrapie prions have been cloned in tg338 transgenic mice (Vilotte *et al.*, 2001) expressing the VRQ allele of ovine PrP. The 263K prion strain corresponds to the historical hamster-adapted scrapie prion (Kimberlin and Walker, 1977). For all prion strains tested, the stocks of infected brain homogenates (20% wt/vol. in 5% glucose) were prepared using at least 3 infected mice at the terminal stage of disease and were aliquoted before storage at -80°C.

A human BAC clone (graciously provided by M. Sarov, Max Planck Insitute, Desden, Germany (Poser *et al.*, 2008)) encompassing the entire *PRNP* Val129 allele was linearized by N*otI* and gel purified prior to microinjection into FVB/N *Prnp*-knockout mouse eggs, as previously described (Vilotte et al., 2001). The transgenic offspring was identified by tail-genomic DNA PCR analysis, and this founder mouse reproduced to derive the *PRNP*-129VV transgenic line (to be published elsewhere).

# Dilution in PMCA buffer & sedimentation velocity fractionation

The 10% PrP^0/0^ brain lysate was prepared using cold PMCA buffer (50 mM Tris-HCl, pH 7.4, 5 mM EDTA, 300 mM NaCl, 1% Triton X-100) and homogenized with a Dounce (Moudjou *et al.*, 2013). The lysate was clarified by centrifugation at 1,000 × *g* for 2 min at 4°C and the resulting supernatant, was aliquoted, and stored at −80°C. To explore the effect of dilution into PMCA buffer on PrP^Sc^ quaternary structure, 20% brain homogenate of 263K, LA21K *fast* or sCJD-MM1 were diluted in 10% w/v PrP^0/0^ brain lysate at different ratio: not diluted, 1:3, 1:5, 1:15, 1:20 dilution. After 4h incubation at 4°C in the absence of sonication, the quaternary structure of PrP^Sc^ assemblies was investigated using sedimentation velocity technique as previously described (Tixador *et al.*, 2010). Briefly, immediately after the 4 hours incubation, the sample was solubilized by adding an equal volume of solubilization buffer (50 mM HEPES pH 7.4, 300 mM NaCl, 10 mM EDTA, 2 mM DTT, 4% wt/vol dodecyl- β-D-maltoside (Sigma)) and incubated for 45 min on ice. Sarkosyl (N-lauryl sarcosine; Fluka) was added to a final concentration of 2% wt/vol and incubated for 30 min on ice. 150 µl of solubilized samples was carefully loaded on a 4.8 ml continuous 10–30% iodixanol gradient (Optiprep, Sigma-Aldrich), with a final concentration of 25 mM HEPES pH 7.4, 150 mM NaCl, 2 mM EDTA, 0.5% Sarkosyl. The gradients were centrifuged at 285 000 x *g* for 45 min in a swinging-bucket SW-55 rotor using an Optima LE-80K ultracentrifuge (Beckman Coulter) and 30 fractions were manually collected from the bottom using a peristaltic pump. The collected fractions were proteinase K (PK) digested at the final concentration of 50µg/ml and analysed by western blotting using a biotinylated anti-PrP Sha31 monoclonal antibodyafter migration using 12% Bis-Tris Criterion gels (Bio-Rad, Marne la Vallée, France). Immunoreactivity was visualized by chemiluminescence (GE Healthcare) after acquisition of chemiluminescent signals with a Chemidoc digital imager (Bio-Rad, Marnes-la-Coquette, France).

## Stainless steel wire contamination

5mm long segments were cut from stainless steel wire (ASI 316 FE 245105-Goodfellow, England; diameter 0.25-0.30 mm) and ultrasonically cleaned for 15min (model VTUSC2, frequency 42000 Hz) in pure ethyl acetate then rinsed once in a 2-propanol bath and twice in milli-Q water. The wires were again ultrasonically treated in a 2% Triton X100 solution for15min before being rinsed 3 times with milli-Q water and then dried for 16h under a laminar flow hood. The cleaned wires were contaminated in batches of 5 wires in a 2ml Eppendorf tube containing 200µl of 10% m/v brain homogenate (corresponding to dilution point 10^-1^). The other points of the range (10^-2^ to 10^-9^) were obtained by 10-fold serial dilutions of 10% positive brain homogenate in 10% brain homogenate from a uninfected animal before addition of wires. After 1h incubation at 22°C under slow agitation (400 rpm, Thermomixer, Eppendorf), the homogenate was removed by pipetting and the wires were dried in a petri dish for 16h under a laminar flow hood taking care that all wires were individualized. After complete drying, the wires were distributed in batches of 5 in 2ml Eppendorf tubes and rinsed three times with 1ml PBS 1X (Sigma) for 5min at room temperature and under slow agitation (400rpm). The wires were again individualized in a petri dish and left to dry for 2h under a laminar flow hood.

## Miniaturized bead-PMCA

The specific templating activity of the different prion strains was measured using the mb-PMCA technique (Moudjou *et al.*, 2013). Briefly, serial ten-fold dilutions of the initial 20% brain homogenate were immediately mixed with the brain lysate (10% wt/vol.) from healthy BacV mice containing 1% of Sodium Dextran Sulfate (DSS > 500 kDa, Sigma Aldrich, Saint Quentin Fallavier, France) in presence of one Teflon bead. One PMCA round of 96 cycles (30 s sonication at 220- 240 Watts, followed by 29.5 min of incubation at 37°C) was performed in a 96-well microplate, using a Q700 sonicator (Misonix, Farmingdale, NY, USA, and Delta Labo, Colombells, France). Aliquots of the amplified samples were PK digested at a final concentration of 115 µg/ml with 0.6% SDS for 1 h at 37°C prior to immunoblot analysis, as described above.

In order to detect prion adsorbed to a stainless-steel surface, wires previously contaminated with serial 10-fold dilutions were directly inserted in a 96-well microplate already loaded with one Teflon bead and 40µL of BacV-brain lysate per well. After one PMCA round of 96 cycles (30 s sonication at 220- 240 Watts, followed by 29.5 min of incubation at 37°C), 4µL of each well were transferred in a new 96-well microplate previously loaded with 36µL of BacV-brain lysate and one Teflon beads. This new microplate was subjected to a second PMCA round of 96 cycles prior immunoblot analysis.

References

Béringue, V. *et al.* (2008) ‘Transmission of atypical bovine prions to mice transgenic for human prion protein’, *Emerging Infectious Diseases*. Emerg Infect Dis, 14(12), pp. 1898–1901. doi: 10.3201/eid1412.080941.

Jaumain, E. *et al.* (2016) ‘Absence of Evidence for a Causal Link between Bovine Spongiform Encephalopathy Strain Variant L-BSE and Known Forms of Sporadic Creutzfeldt-Jakob Disease in Human PrP Transgenic Mice’, *Journal of Virology*. American Society for Microbiology, 90(23), pp. 10867–10874. doi: 10.1128/jvi.01383-16.

Kimberlin, R. H. and Walker, C. A. (1977) ‘Characteristics of a short incubation model of scrapie in the golden hamster’, *Journal of General Virology*. J Gen Virol, 34(2), pp. 295–304. doi: 10.1099/0022-1317-34-2-295.

Moudjou, M. *et al.* (2013) ‘Highly infectious prions generated by a single round of microplate- based protein misfolding cyclic amplification’, *mBio*. mBio, 5(1). doi: 10.1128/mBio.00829-13.

Poser, I. *et al.* (2008) ‘BAC TransgeneOmics: A high-throughput method for exploration of protein function in mammals’, *Nature Methods*. Nat Methods, 5(5), pp. 409–415. doi: 10.1038/nmeth.1199.

Tixador, P. *et al.* (2010) ‘The physical relationship between infectivity and prion protein aggregates is strain-dependent.’, *PLoS pathogens*, 6(4), p. e1000859. doi: 10.1371/journal.ppat.1000859.

Vilotte, J.-L. *et al.* (2001) ‘Markedly Increased Susceptibility to Natural Sheep Scrapie of Transgenic Mice Expressing Ovine PrP’, *Journal of Virology*. American Society for Microbiology, 75(13), pp. 5977–5984. doi: 10.1128/jvi.75.13.5977-5984.2001.
